# Post-translational regulation of sucrose transporters by direct protein–protein interactions

**DOI:** 10.3389/fpls.2013.00237

**Published:** 2013-07-02

**Authors:** Undine Krügel, Christina Kühn

**Affiliations:** ^1^Institute of Plant Biology, University of Zürich, ZürichSwitzerland; ^2^Department of Plant Physiology, Institute of Biology, University of BerlinBerlin, Germany

**Keywords:** protein–protein interaction, sucrose allocation, membrane microdomains, detergent-resistant membrane fraction, subcellular trafficking

## Abstract

Sucrose transporters are essential membrane proteins for the allocation of carbon resources in higher plants and protein–protein interactions play a crucial role in the post-translational regulation of sucrose transporters affecting affinity, transport capacity, oligomerization, localization, and trafficking. Systematic screening for protein interactors using sucrose transporters as bait proteins helped identifying several proteins binding to sucrose transporters from apple, *Arabidopsis*, potato, or tomato using the split ubiquitin system. This mini-review summarizes known sucrose transporter-interacting proteins and their potential function in plants. Not all of the identified interaction partners are postulated to be located at the plasma membrane, but some are predicted to be endoplasmic reticulum-residing proteins such as a protein disulfide isomerase and members of the cytochrome b5 family. Many of the SUT1-interacting proteins are secretory proteins or involved in metabolism. Identification of actin and actin-related proteins as SUT1-interacting proteins confirmed the observation that movement of SUT1-containing intracellular vesicles can be blocked by inhibition of actin polymerization using specific inhibitors. Manipulation of expression of these interacting proteins represents one possible way to modify resource allocation by post-translational regulation of sucrose transporters.

## INTRODUCTION

Elucidation of the interactome of membrane proteins seems to be a powerful tool to gain insights into signal transduction and regulation of nutrient transport in higher plants. For *Arabidopsis*, a robotic screening method was established based on the split ubiquitin system in yeast (Stagljar et al., 1998) in order to systematically analyze the membrane-based interactome with potential application in fungi, plants, and metazoan (Lalonde et al., 2010). This review will rather focus on single protein–protein interactions (PPIs) of plant sucrose transporters and summarize recent research in this field published during the past 5 years.

## CO-LOCALIZED SUCROSE TRANSPORTERS IN THE PHLOEM CAN INTERACT WITH EACH OTHER

Sucrose transporters from potato and tomato belonging to different phylogenetic clades (Kühn and Grof, 2010) co-localize in phloem sieve elements (SEs;Barker et al., 2000;Weise et al., 2000;Reinders et al., 2002a). Using the split ubiquitin system it was shown that SlSUT1, SlSUT2, and SlSUT4 from tomato (*Solanum lycopersicum*) are able to form homo- and heterooligomers (Reinders et al., 2002a) and, even when expressed separately from different plasmids, the two halves of SlSUT1 were able to reconstitute a functional transporter (Reinders et al., 2002b).

Controversial localization of *Arabidopsis* sucrose transporters has been reported. Whereas AtSUC2 is assumed to be localized in phloem companion cells (CCs;Stadler and Sauer, 1996), AtSUC3/SUT2 was finally localized in phloem SEs (Meyer et al., 2004), and AtSUT4 was also detected in mesophyll protoplasts (Endler et al., 2006). Nevertheless, the promoter activity of the genes encoding all three sucrose transporters was mainly detected in phloem CCs (Schulze et al., 2003). This is not unexpected, since SE-specific sucrose transporter (SUT) expression is efficiently inhibited by help of a companion-cell-specific antisense construct (Kühn et al., 1996). *Arabidopsis* SUTs interact with each other in yeast cells (Schulze et al., 2003). Detection of strong promoter activity of AtSUT4 in phloem minor veins (Weise et al., 2000;Schulze et al., 2003) and the detection of AtSUT4 transcripts in mesophyll cells (Endler et al., 2006) argue for the presence of AtSUT4 in both tissues.

**FIGURE 1:**
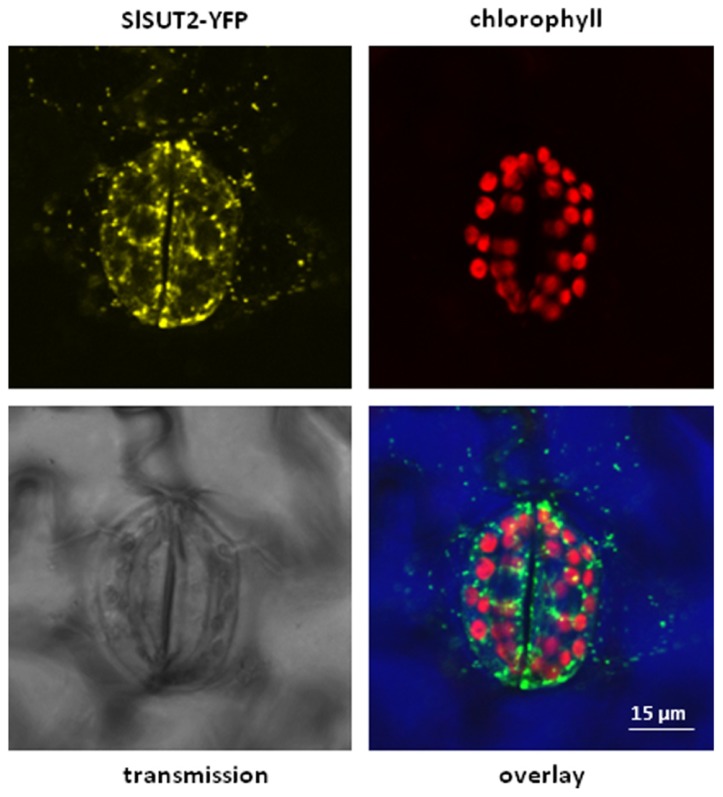
**Overlay picture showing subcellular distribution of a SlSUT2-YFP construct expressed under control of the constitutive CaMV 35S promoter in the vector pK7YWG2.0 (Karimi et al., 2002) in stably transformed tobacco plants (*Nicotiana tabacum*)**. SlSUT2-YFP fluorescence is mainly retained in intracellular structures, a possible argument for its non-functionality at the plasma membrane. Contrast of the overlay picture was enhanced by choosing the RGB mode leading to color modification. YFP fluorescence is therefore shown in green, chlorophyll autofluorescence is shown in red. Color of the transmission image is shown in blue.

It should be also noted that the subcellular localization of SUT/SUCs does not completely overlap with each other. It is obvious that the subcellular localization of sucrose transporters is crucial for their functionality. Whereas StSUT1 localization is primarily at the plasma membrane (PM), SlSUT2 is also detectable in intracellular structures (Figure 1). SlSUT2 and StSUT1 interact completely different populations of proteins (Krügel and Kühn, unpublished results) suggesting different functions. The fact that SlSUT2–YFP fusion mainly localizes to intracellular membranes in stably transformed tobacco plants (Figure 1) might be one reason for its non-functionality as sucrose transporter in heterologous expression systems. It should be taken into consideration also for members of the SUT4 family that their subcellular localization is not necessarily static and confined to one single compartment (Chincinska et al., 2013), but undergoes dynamic changes during development, degradation or initial targeting to two different compartments.

The ability of StSUT1 to form dimers was confirmed experimentally by biochemical methods such as blue native PAGE, chemical cross-linking, two-dimensional gel electrophoresis, and bimolecular fluorescence complementation (BiFC;Krügel et al., 2008). Heterologous expression of StSUT1 from *Solanum tuberosum* revealed redox-dependent formation of homodimers (Krügel et al., 2008). The capacity of StSUT1 and StSUT4 to form heterodimers *in planta* was confirmed by BiFC where heterodimers were mainly detected in the endoplasmic reticulum (ER;Krügel et al., 2012). Thus heterodimer formation between SUTs can be found even when their subcellular localization does not completely overlap.

## SUT1-INTERACTING PROTEINS

The yeast split ubiquitin system has been used to systematically scr-een for StSUT1-interacting proteins and seven different candidate proteins have been identified: a PDI, an enolase, an aldehyde dehydrogenase, a proton pyrophosphatase, an aquaporin, Snakin-1 (SN1), and an unknown protein (Krügel et al., 2012).

The attempts to confirm these interactions by co-immunoprecipitation (Co-IP) using specific antibodies or commercially available monoclonal antibodies raised against tagged and over-expressed SoSUT1 protein were not successful (Krügel et al., 2012), but large overlap was observed between proteins co-precipitated with StSUT1 and the detergent-resistant membrane (DRM) fraction of potato source leaf plasma membranes.

Immunoprecipitation was performed with either PMs from potato wild-type plants using an affinity-purified peptide antibody or alternatively with PMs from potato plants over-expressing a c-myc-tagged version of the sucrose transporter SoSUT1 from spinach (Leggewie et al., 2003) using monoclonal antibody against c-myc tag. Two candidates interact with StSUT1 from potato as well as with the over-expressed SoSUT1 from *Spinacia oleracea*: a plasma membrane H^+^-ATPase (CAA54045) and a temperature-induced lipocalin (TIL; ABB02386). TILs are discussed to be involved in freezing tolerance of plants and/or in membrane stability and abiotic stress responses (Charron et al., 2005;Chi et al., 2009).

### PROTEINS INVOLVED IN SUBCELLULAR PROTEIN TARGETING

StSUT1 was shown to undergo endocytosis in response to brefeldin A treatment and to be present in intracellular vesicles recycled at the PM. Motility of these vesicles is efficiently abolished by application of actin polymerization inhibitors such as latrunculin and actinomycin D (Liesche et al., 2010). This strongly suggests that movement of StSUT1-containing vesicles occurs in an actin-dependent manner involving the cellular cytoskeleton. Direct interaction of StSUT1 with actin and actin-related proteins strongly supports this hypothesis. The actin-related protein ARPC1/ARBC1A which was co-precipitated with StSUT1 (Krügel et al., 2012) is a component of the Arp2/3 complex implicated in the control of actin polymerization (Harries et al., 2005).

Also other StSUT1-interacting proteins identified by Co-IP are obviously involved in subcellular protein targeting and endosomal recycling at the PM. The integral membrane protein of the Yip1 family was shown to interact with the spinach SoSUT1 and is assumed to play a role in membrane trafficking. Yip1 family proteins are required for the biogenesis of ER-derived COPII vesicles in yeast and mammalian cells (Heidtman et al., 2005;Lorente-Rodriguez et al., 2009).

Other StSUT1-interacting proteins identified by Co-IP make part of the secretory pathway: Sec61 (NP_177993) is an ER-localized heterotrimeric protein translocator involved in the transport of proteins into and out of the ER (Rapoport, 1992) and mediates the export of misfolded proteins to the cytoplasm for degradation in various organisms (Pilon et al., 1997;Schmitz et al., 2000). Sec34 (NP_177485) is well known to regulate vesicular trafficking from ER to Golgi (Loh and Hong, 2002) and might be responsible for correct secretion of SUT1 to the plasma membrane.

### SUT1-INTERACTING PROTEINS INVOLVED IN SIGNALING

Many other StSUT1-interacting proteins that have been identified either by SUS or by Co-IP are metabolic enzymes or involved in signaling or both.

The yeast Gal83 protein (CAB52141) is an important player under glucose limitation conferring specificity of the Snf1 complex to target proteins such as transcription factors (Yang et al., 1994). A correlation between Gal83 expression and assimilate transport in plants was described (Schwachtje et al., 2006).

A similar regulation of StSUT1 via phosphorylation as described for monosaccharide transporters in yeast can therefore be assumed (Carlson, 1998). Experimental data support the hypothesis of regulation of sucrose transporter activity via phosphorylation (Roblin et al., 1998;Nühse et al., 2004;Niittyla et al., 2007). 14-3-3 proteins are described to regulate many cellular processes by binding to phosphorylated proteins. The interaction of *Arabidopsis* 14-3-3 isoforms in response to changes in the plant nutrient status helped to identify new targets involved in nitrogen and sulfur metabolism (Shin et al., 2011). Among other sugar and mannitol transporters, AtSUC6 from *Arabidopsis* directly interacts with a 14-3-3χ protein of *Arabidopsis* suggesting that AtSUC6 is phosphorylated as well (Shin et al., 2011).

The detailed function of SUT phosphorylation remains to be elucidated. It is still unclear whether or not phosphorylation of the StSUT1 protein affects its subcellular localization or dimerization behavior.

### METABOLISM

Adenosine diphosphate (ADP)-glucose pyrophosphorylase (AGPase) is the key enzyme in starch biosynthesis and forms an allosteric heterotetramer. The activity of AGPase is regulated, e.g., by sugars and light through post-translational redox-dependent dimerization of the small subunit (Hendriks et al., 2003;Geigenberger et al., 2005).

Furthermore it is known from transgenic plants with reduced expression of plant SUTs that starch levels in source leaves are dramatically increased, which can be visualized macroscopically (Bürkle et al., 1998;Hackel et al., 2006) or by electron microscopy (Bürkle et al., 1998;Schulz et al., 1998;Chincinska et al., 2008). The identification of StSUT1-interacting proteins by Co-IP revealed direct interaction of StSUT1 with glucose-1-phosphate adenyltransferase (CAA53741) which represents the large subunit of the AGPase tetramer (Krügel et al., 2012). Therefore adaptation of starch biosynthetic capacity is not only possible via sugar availability and the redox-dependent dimerization of the small AGPase subunit but also by direct PPI between StSUT1 and the large subunit of AGPase.

### REDOX-REGULATION: SNAKIN-1, PDI

One of the interaction partners of StSUT1 repeatedly identified in several independent screens was SN1 (Krügel et al., 2012). SN1 is a small cysteine-rich cell wall protein from *Solanum tuberosum* with an assumed antimicrobial function. It belongs to the Snakin/GASA (gibberellic acid stimulated in *Arabidopsis*) protein family and its physiological function was recently elucidated by generation of transgenic potato plants (Nahirnak et al., 2012a,b). SN1 is a PM protein and silencing of SN1 expression in transgenic potato plants led to reduced plant height and leaf size, whereas the mean cell size was increased. SN1 silencing affects cell division, cell wall composition, and leaf primary metabolism, i.e., the level of intermediates of the tricarboxylic acid (TCA) cycle. Redox homeostasis of SN1-silenced plants seems to be disturbed because the level of reactive oxygen species (ROS) is increased in these plants, while the level of ascorbate is reduced (Nahirnak et al., 2012b).

Computational prediction suggests that conserved oxidized cysteines in Snakin/GASA proteins can create up to five disulfide bridges that may act catalytically, i.e., playing a role in redox regulation exhibiting antioxidant activity (Wigoda et al., 2006). In *Arabidopsis*, the GASA4 protein affects the level of H_2_O_2_ and NO upon wounding and participates in GA-dependent signaling of flowering induction (Rubinovich and Weiss, 2010).

It is assumed that OsGSR1, a Snakin/GASA protein from rice, plays an important role in the cross-talk of GA and brassinosteroids (BR) signaling pathways (Wang et al., 2009). The expression of OsGSR1 is induced by gibberellins (Gas) and repressed by BR. Thus, members of the SN1/GASA protein family cover aspects in plant development, beyond their assumed function in response to biotic or abiotic stresses (Nahirnak et al., 2012b).

The physiological role of StSUT1–SN1 interaction in potato remains to be elucidated. A similar phenotype as in SN1-inhibited potato is observed when the expression of another SUT-interacting protein, the protein disulfide isomerase, is inhibited in transgenic potato plants by RNA interference (RNAi) silencing (E. Eggert and C. Kühn, unpublished results). In view of the fact that StSUT1 forms dimers in a redox-dependent manner (Krügel et al., 2008) it is interesting to note that two out of seven StSUT1-interacting proteins identified by SUS seem to be involved in redox homeostasis.

## INTERACTORS OF SUT4 MEMBERS: MdSUT1 AND AtSUT4 INTERACT WITH PROTEINS OF THE Cyb5 FAMILY

The MdSUT1 protein of *Malus domestica,* belonging to the SUT4 subfamily, was immunolocalized to the plasma membrane of apple phloem and parenchyma cells. A systematic SUS screen was performed using MdSUT1 and the sorbitol transporter MdSOT6 as bait proteins (Fan et al., 2009). Cytochrome b5 (Cyb5) was identified several times independently as interaction partners of MdSUT1 and of MdSOT6 (Table 1).

**Table 1:** Protein–protein interaction partners of plant sugar transporters identified by the yeast two hybrid split ubiquitin system.

Bait protein	Localization	Prey proteins	Confirmation	Reference
StSUT1 (*Solanum tuberosum*, SUT1 clade)	PM	Protein disulfide isomerase (PDI)	GST pull-down, BiFC, DRM protein, FRET acceptor bleaching, also interacts with SlSUT2 and StSUT4	Krügel etal. (2012)
		Snakin-1	Several times (>5) in independent screens
		Inorganic pyro-phosphatase (PPi)	DRM protein
		Tonoplast intrinsic protein (TIP)	DRM protein
		Enolase	not confirmed
		Aldehyde de-hydrogenase (ADH)	Several times in independent screens
		Unknown protein	not confirmed
MdSUT1 (*Malus domestica*, SUT4 clade)	PM	Cytochrome b5 (Cyb5)	BiFC, Co-IP, identified in 17 independent colonies	Fan etal. (2009)
MdSOT6 (*Malus domestica*, sorbitol transporter)	PM	Cyb5	BiFC, Co-IP, identified in 20 independent colonies	Fan etal. (2009)
AtSUT4 (*Arabidopsis thaliana*, SUT4 clade)	vacuole	Cyb5-1	BiCF, Co-IP	Li etal. (2012)
		Cyb5-2
		Cyb5-3
		Cyb5-4
		Cyb5-6

Cyb5s are small membrane-anchored proteins with a putative heme/steroid binding domain. They are involved in a number of oxidative reactions. Interaction between MdSUT1 and Cyb5 was confirmed by immunoprecipitation and BiFC. Since MdSUT1 was localized to the PM whereas Cyb5 is expected in the ER, electron microscopic immunolocalization was performed showing accurately that Cyb5 is localized in ER cisternae, which are connected to the PM (Fan et al., 2009).

β-Galactosidase (lacZ) activity tests in yeast have been used to quantify the strength of interaction in the presence or absence of glucose or sucrose in the medium. Increasing sucrose concentrations inhibited MdSUT1 interaction with Cyb5, whereas varying concentrations of glucose (which is not transported by MdSUT1) did not affect the strength of interaction (Fan et al., 2009).

Deletion constructs of the SUT-interacting Cyb5 revealed that the C-terminal region containing the membrane spanning domain of Cyb5 is essential for the interaction with sugar transporters, whereas the N-terminus only plays a role in the strength of the interaction. Thus, it is likely that the C-terminal part of Cyb5 interacts with the N-terminal part of MdSUT1 and that interaction takes place within the membrane. Co-expression of the Cyb5 protein with MdSUT1 in yeast increased affinity of MdSUT1 toward the substrate sucrose. Besides inhibiting PPI substitution of leucine_73_ with proline abolished this increase in affinity. It was suggested that the sugar transporter–Cyb5 complex, which is strengthened under sugar starvation, promotes sugar uptake when sugar availability is limited.

In *Arabidopsis*, the same authors showed that AtSUT4, but not homologous transporters in the SUT1 or SUT2 subfamily, is able to interact with five different members of the Cyb5 family. Confirmation of interaction was obtained from pull-down assays and via BiFC (Li et al., 2012). Both *atsut4* and *cyb5-2* mutant plants show decreased sensitivity toward sucrose and glucose with respect to seed germination and it is suggested that the AtSUT4/Cyb5-2 complex might be involved in sensing or signaling not only of sucrose but also of glucose (Li et al., 2012).

## SUMMARY AND OUTLOOK

### PITFALLS

The identification of StSUT1-interacting proteins by different methods revealed completely different populations of interacting proteins. This is possibly explained by the differences of the two methods that have been used: for split ubiquitin screens, often a complete cDNA library is screened. In case of the StSUT1 split ubiquitin screen, the potato cDNA library was generated from RNA isolated and pooled from various tissues and developmental stages (Krügel et al., 2012). Thus, PPIs were demonstrated which under native conditions are not possible due to expression in different tissues, cells, developmental stages, and/or cellular compartments. PPIs determined using this method always need to be confirmed *in planta* in order to eliminate false-positive interaction partners.

Immunoprecipitation experiments, on the other hand, have been performed with plant extracts containing PM-enriched fractions isolated at a given developmental stage from source or sink leaves (Krügel et al., 2012). These interacting proteins are at least present at the same developmental stage and in the same isolated membrane fraction. Thus, a different population of proteins is expected for this screen of PPIs. Both sets of candidate genes identified with the two different methods need further confirmation.

### THE POTENTIAL ROLE OF SUBCELLULAR COMPARTMENTATION

Elucidation of the StSUT1 interactome helped to get information about the regulation and subcellular localization of this SUT. Its presence in the DRM fraction was confirmed by liquid chromatography-tandem mass spectrometry (LC-MS/MS;Krügel et al., 2012) and its association to lipid raft-like microdomains was visualized by expression of green fluorescent protein (GFP) fusion constructs in yeast (Krügel et al., 2008). SUT1 is constantly recycled at the SE PM in an actin-dependent manner (Liesche et al., 2010). Identification of actin-related proteins and components of the secretory pathway such as Sec34 and Sec61 among the StSUT1-interacting partners support these findings. A large overlap was observed between StSUT1-interacting proteins identified by Co-IP and proteins showing reduced detergent solubility (Krügel et al., 2012).

The question is now whether association of the StSUT1 to membrane microdomains is a prerequisite for endocytosis and recycling. Methyl-β-cyclodextrin (MβCD) causes sterol depletion of the plant PM and thereby inhibits raft formation (Roche et al., 2008). The significantly increased activity of the glucose transporter GLUT1 in the presence of MβCD is explained by inhibition of endocytosis thereby increasing the number of transporters at the PM (Barnes et al., 2004;Caliceti et al., 2012). It is indispensable to test the impact of MβCD on the activity of SUT1, whose endocytosis seems to be inhibited by MβCD similarly to GLUT1 in the mammalian system (Liesche et al., 2010).

Raft association of plant membrane proteins may also impact lateral segregation. The potassium channel AtKAT1 was localized to microdomains and a low lateral mobility was observed (Reuff et al., 2010). Cholesterol depletion of the membrane decreases lateral mobility of PM proteins (Kwik et al., 2003) suggesting that association to raft-like microdomains enhance lateral diffusion.

Interestingly, the plasma membrane lining plasmodesmata share characteristics with membrane rafts, since typical raft proteins such as glycosylphosphatidylinositol (GPI)-anchored proteins, the callose binding protein, remorin and also sphingolipids and phytosterols are enriched in the PM of plasmodesmata (Mongrand et al., 2010).

Translation of StSUT mRNAs is assumed to take place in CCs and the proteins need to be targeted to the PM of neighboring SEs (Kühn and Grof, 2010). Increased lateral mobility due to raft-like properties of the PM lining plasmodesmata might represent one possible way to facilitate correct targeting of SUTs from CC into enucleate SEs.

A recent paper, however, investigated lateral mobility of minimal membrane proteins by fluorescence recovery after photobleaching (FRAP) and points to the important role of the cell wall in immobilizing PM proteins, whereas association to membrane microdomains is assumed to play only a minor role in lateral mobility (Martiniere et al., 2012).

### SIGNALING

Raft-like membrane compartments are regarded as organizing principle allowing attribution to protein-based sub-organellar compartments within a single cell to facilitate enzymatic reactions or cellular dynamics (Holthuis and Ungermann, 2012). Moreover, lipid rafts form cholesterol-enriched redox signaling membrane platforms providing an important driving force, e.g., for the assembly of nicotinamide adenine dinucleotide phosphate (NADPH) oxidase subunits to form (or activate) a functional enzyme complex in the PM (Jin et al., 2011). Depending on the cell type, sterol depletion of the PM using MβCD either delays NADPH oxidase activation leading to decreased ROS production (Vilhardt and van Deurs, 2004), decreased NADPH oxidase activity (Rao Malla et al., 2010), or promotes activation of NADPH oxidase (Han et al., 2008).

Lipid rafts are assumed to represent signaling platforms perhaps enabling membrane proteins to get in close contact to signaling proteins, kinases, phytohormone receptors, etc. However, the question remains whether the redox-dependent dimerization of SUT proteins and phosphorylation/dephosphorylation events are facilitated if the protein is concentrated in raft-like microdomains.

Manipulation of expression of SUT-interacting proteins represents a powerful tool to impact sucrose transport activity at the post-translational level in future experiments thereby strongly affecting carbon partitioning and resource allocation within crop plants.

## Conflict of Interest Statement

The authors declare that the research was conducted in the absence of any commercial or financial relationships that could be construed as a potential conflict of interest.

## Abbreviations

BIFC: bimolecular fluorescence complementation
CC: companion cell
DRM: detergent–resistant membranes
MS: mass spectrometry
PDI: protein disulfide isomerase
PM: plasma membrane
PPI: protein–protein interaction
SE: sieve element
SUC: sucrose carrier
SUS: split ubiquitin system
SUT: sucrose transporter
